# Polythiophene/Ti_3_C_2_T_X_ MXene Composites for Effective Removal of Diverse Organic Dyes via Complementary Activity of Adsorption and Photodegradation

**DOI:** 10.3390/molecules30061393

**Published:** 2025-03-20

**Authors:** Young-Hwan Bae, Seongin Hong, Jin-Seo Noh

**Affiliations:** 1 Department of Semiconductor Engineering, Gachon University, 1342 Seongnam-daero, Sujeong-gu, Seongnam-si 13120, Gyeonggi-do, Republic of Korea; byh2696@gmail.com; 2 Department of Physics, Gachon University, 1342 Seongnam-daero, Sujeong-gu, Seongnam-si 13120, Gyeonggi-do, Republic of Korea

**Keywords:** Ti_3_C_2_T_X_ MXene, polythiophene nanospheres, dye removal, adsorption, photocatalyst

## Abstract

This study presents an effective method to remove organic dyes from wastewater, using a composite of few-layered porous (FLP) Ti_3_C_2_T_x_ MXene and polythiophene (PTh) nanospheres. The FLP MXene, which was pre-synthesized by a series of intercalation, heat-induced TiO_2_ formation, and its selective etching, was combined with PTh nanospheres via a simple solution method. The composite effectively removed various organic dyes, but its efficiency was altered depending on the type of dye. Particularly, the removal efficiency of methylene blue reached 91.3% and 97.8% after irradiation for 10 min and 1 h, respectively. The high dye removal efficiency was attributed to the large surface area (32.01 m^2^/g) of the composite, strong electrostatic interaction between the composite and dye molecules, and active photodegradation process. The strong electrostatic interaction and large surface area could facilitate the adsorption of dye molecules, while photocatalytic activity further enhance dye removal under light. These results are indicative that the PTh/FLP MXene composite may be a promising material for environmental remediation through synergistic processes of adsorption and photocatalysis.

## 1. Introduction

Synthetic dyes, including Methylene Blue (MB), Rhodamine B (RhB), and Orange G (OG), are widely used in industries, such as textiles, pharmaceuticals, and food production [1,2]. Despite their widespread use, these dyes pose serious environmental concerns primarily due to their complex molecular structures, which make them resistant to biodegradation [3,4]. The toxicity and carcinogenicity of synthetic dyes threaten aquatic life and human health, necessitating the development of efficient remediation technologies for removing them from wastewater [5,6]. These dyes not only reduce water quality by preventing sunlight penetration but also bioaccumulate in aquatic organisms, leading to long-term ecological disruptions [7]. Traditional wastewater treatment techniques, such as coagulation, flocculation, and chemical oxidation, often prove inadequate for the complete removal of these pollutants [8,9]. Coagulation and flocculation primarily convert dyes into sludge, creating a secondary disposal issue without fully breaking down the toxic compounds [10]. Emerging methods, like photocatalysis, offer promising alternatives by harnessing advanced materials to break down pollutants into less harmful compounds [11,12]. Photocatalysis is an advanced oxidation process that utilizes light energy to activate a photocatalyst, which can generate electron-hole pairs or facilitate other oxidative mechanisms, such as those employed in Fenton-like catalysis, to degrade pollutants [13,14]. This process is particularly effective for degrading complex organic molecules, like synthetic dyes. However, finding suitable photocatalysts with high efficiency and stability under light exposure remains a critical challenge in environmental science [11].

MXenes, a class of two-dimensional (2D) transition metal carbides or nitrides, have garnered significant attention in recent years due to their unique properties, including high surface area, electrical conductivity, and tunable surface chemistry [15,16]. The excellent hydrophilicity and abundant surface functional groups (–OH, =O, –F) of MXenes make them ideal for water treatment applications employing photocatalysis and adsorption [9,17]. The layered structure of MXenes provides ample sites for pollutant adsorption, while their high electrical conductivity enhances charge transfer in photocatalytic processes [5]. Studies have demonstrated that MXene-based composites can achieve over 90% degradation efficiency for dyes, like MB and RhB, in 60 min [6,14,18]. However, conventional MXenes have multilayer structures due to the van der Waals interlayer force, which reduce their surface area and surface functionality [19]. As a potential solution to this problem, various conducting polymers were introduced into MXenes, including polyaniline (PANI), polypyrrole (PPy), and polythiophene (PTh) [20]. Of those, polythiophene (PTh) is a well-known conducting or semiconducting polymer that has gained significant attention due to its π-conjugated electron structure, which may facilitate charge separation and photon absorption during photocatalytic processes [2,17]. The PTh is widely studied for its use in solar cells, sensors, and environmental remediation technologies [10]. However, PTh alone often faces challenges, such as limited photocatalytic efficiency and reduced surface area, which necessitates its combination with other materials. The combination of MXenes with PTh has emerged as a powerful strategy to enhance both the photocatalytic and adsorptive properties of these materials [5,20]. MXenes offer high surface area and dense surface functional groups for pollutant adsorption, while PTh enhances photocatalytic activity under light irradiation [9,13]. The integration of MXenes and PTh can maximize light absorption while enhancing electron mobility, providing a distinct advantage over traditional photocatalysts [11,12]. In actuality, several studies have demonstrated the ability of MXene/PTh composites to degrade over 95% of organic dyes, like MB and RhB, under UV light [8,14].

Despite the promising potential of MXene/PTh composites, several challenges remain, and further works are required. One of the primary challenges is the stability of MXenes in aqueous environments, as they are prone to oxidation [15]. The total surface areas of a MXene and PTh should be maximized, and the composite should perform well to multiple pollutants. In this work, few-layered porous (FLP) Ti_3_C_2_T_x_ MXene was synthesized by a unique method, and it was hybridized with PTh nanospheres via a simple solution protocol. Enhanced dye removal efficiencies were demonstrated to multiple dyes, and the roles of physical adsorption and photodegradation were elucidated.

## 2. Results and Discussion

### 2.1. Morphologies and Compositions

**Figure 1:**
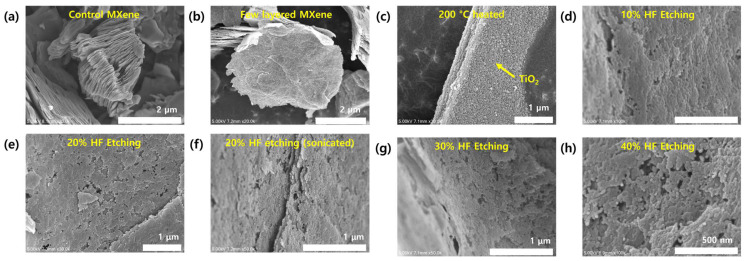
SEM images of (**a**) control Ti_3_C_2_T_X_ MXene, (**b**) few-layered (FL) Ti_3_C_2_T_X_ MXene, (**c**) heat-treated FL MXene at 200 °C, and (**d**–**h**) FLP MXenes etched with different concentrations of HF solutions. The FLP MXene in (**f**) underwent an additional sonication step after 20% HF etching.

**Figure 2:**
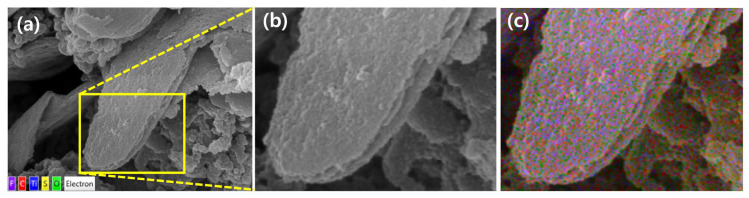

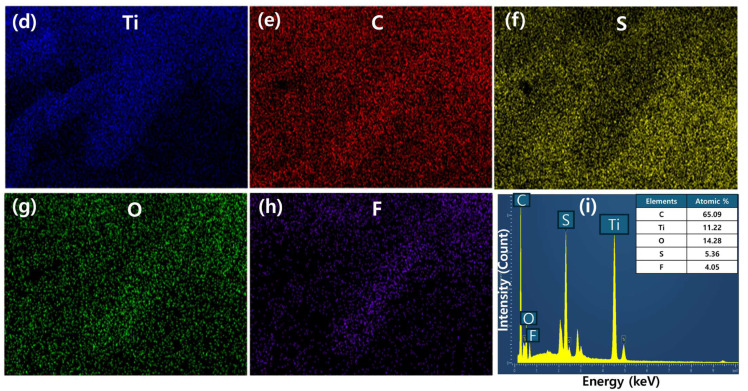
(**a**) SEM image of PTh/FLP MXene composite (1:1). (**b**) Magnified image of the selected area in (**a**). (**c**) Overall element map of the selected area. (**d**–**h**) Element-specific maps: (**d**) Ti, (**e**) C, (**f**) S, (**g**) O, (**h**) F. (**i**) SEM-EDX spectrum with an inset of atomic composition.

**Figure 3:**
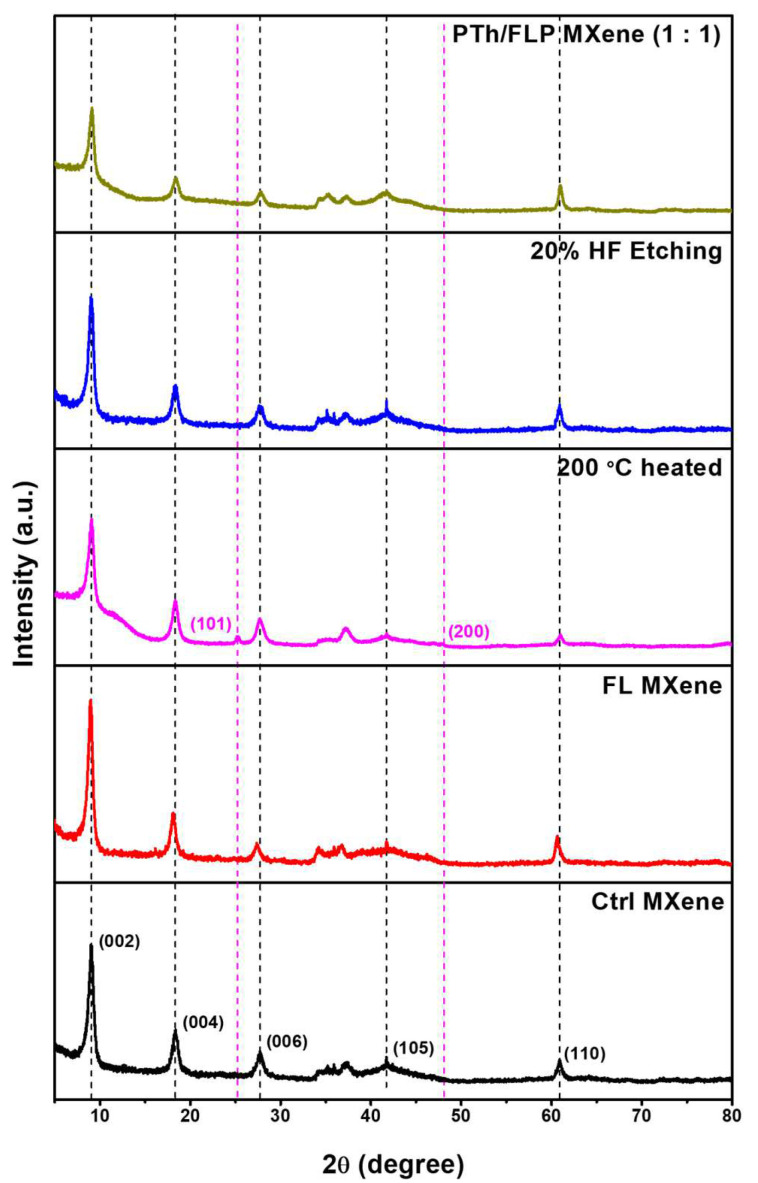
XRD patterns of control MXene (black), FL MXene (red), 200 °C-heated FL MXene (magenta), FLP MXene prepared with 20% HF solution (blue), and PTh/FLP MXene composite with 1:1 weight ratio (dark yellow).

The SEM images of control Ti_3_C_2_T_x_ MXene, few-layered (FL) MXene, heat-treated FL MXene, and FLP MXenes prepared with different HF solutions are shown in Figure 1. The multilayer structure of the control MXene is clearly found in Figure 2a. The number of layers is reduced after the MXene undergoes a delamination process, as seen in Figure 1b, although edges of individual layers become more irregular. Once the FL MXene is heat-treated at 200 °C, TiO_2_ nanoparticles are densely formed on each layer surface by the surface oxidation of the MXene. This phenomenon is consistent with the result previously reported by our group [21], but the nanoparticles in this work look smaller and denser. The TiO_2_ nanoparticles can be completely removed by HF etching [22]. The MXene surface becomes rougher and partially cracked after this HF etching, as clearly observed from Figure 1d–h. The complete removal of TiO_2_ nanoparticles will be further corroborated by the XRD results (Figure 3). It is inferred that plenty of pits on the FL MXene surface treated with 10% HF result from the removal of TiO_2_ nanoparticles since the average pit size is similar to the average nanoparticle size (Figure 1d). However, most pits do not penetrate the MXene flake, which is undesirable for surface area increase and analyte flow. The tendency of vertical penetration and partial cracking becomes stronger as the HF concentration increases. The layer cracking and edge cracking are apparent for FL MXenes treated with 30% and 40% HF solutions, which may arise from excessive mechanical force induced from surplus chemical energy [23]. Thus, FL MXene prepared with 20% HF etching was selected as a standard FLP MXene. Post-sonication turns out to give no additional merit. The SEM images of PTh nanospheres are presented in Figure S1. Well-developed spherical shapes are clearly seen with relatively good size uniformity. Their average size is estimated at ~20 nm.

Figure 2 shows the SEM image and element maps of PTh/FLP MXene composite. Here, the weight ratio between PTh nanospheres and FLP MXene was 1 to 1. From the magnified image in Figure 2b, it is found that PTh nanospheres fully covers the surface of FLP MXene. The dense distribution of PTh nanospheres over the MXene surface is more clearly confirmed from the other image in Figure S2. It is also obvious from Figure S2 that a MXene-rich composite with a weight ratio of 1:5 is lacking PTh nanospheres on the MXene surface. To further analyze the surface components and overall composition, EDX element mapping was performed. As shown in Figure 2d–h, major elements (Ti, C, O, F, S) of MXene and PTh are all observed over the measured area. Particularly, the even and dense distribution of S, which originates only from PTh, represents that PTh nanospheres uniformly cover the MXene flakes (Figure 2f). An integrated dot map for the selected area further supports the even distribution of structural components (Figure 2c). The EDX spectrum and calculated composition for the sample is given in Figure 2i. The overwhelming C content (65.1 at%) relies on the fact that C is the core element for both PTh and Ti_3_C_2_T_x_ MXene. The contents of O and F imply that the surface of FLP MXene is terminated with multiple functional groups, like –OH, =O, and –F.

### 2.2. Crystalline and Bonding Characteristics

Figure 3 exhibits the XRD patterns of control MXene, FL MXene, 200 °C-heated FL MXene, FLP MXene prepared with 20% HF solution, and PTh/FLP MXene composite with a 1:1 weight ratio. For all samples, the major XRD peaks are observed at almost identical positions, and their intensities are comparable to each other, except the PTh/FLP MXene composite. This indicates that the crystal quality of MXene does not deteriorate under the delamination, heat treatment, and subsequent HF etching processes. It is inferred that the slight intensity weakening in PTh/FLP MXene composite was caused by surface-covering and intercalating PTh nanospheres. Actually, three major peaks appear at 2θ = 9.0, 18.3, and 27.6°, which are assigned to (002), (004), and (006) planes of the Ti_3_C_2_T_x_ MXene crystal [24]. Remarkably, TiO_2_ peaks are observed for 200 °C-heated FL MXene, verifying that the surface of FL MXene was locally oxidized by the heat treatment. However, no TiO_2_ peaks are found for FLP MXene. This proves that TiO_2_ nanoparticles pre-formed on the MXene surface were completely removed by HF etching, which is consistent with the previous SEM observations. Meanwhile, no noticeable shift in the (002) peak position is observed after the incorporation of PTh nanospheres, indicating that there was no significant change in the interlayer spacing. This suggests that PTh nanospheres primarily adhere to the surface of MXene rather than interlayers. It is speculated that the interlayer insertion phenomenon of PTh nanospheres observed in Figure S2a,b is not universal, but it rarely occurs particularly for FLP MXene flakes with rather wider gaps.

**Figure 4:**
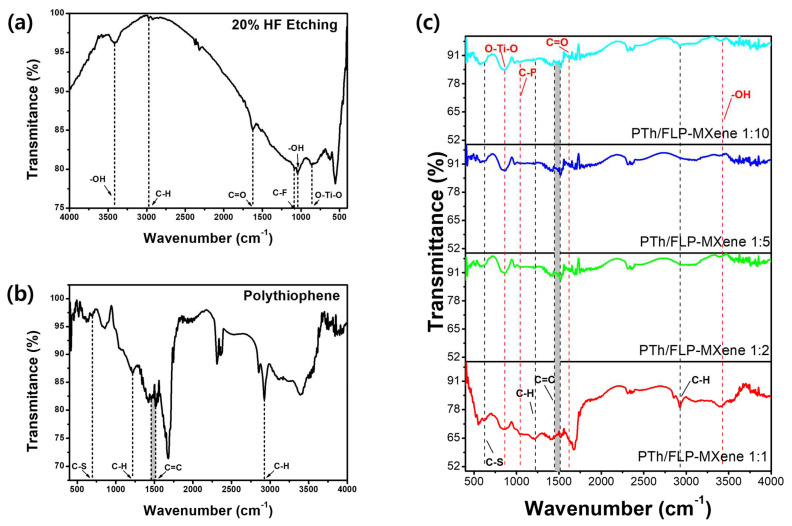
FT-IR spectra of (**a**) FLP MXene prepared with 20% HF solution, (**b**) PTh nanospheres, and (**c**) PTh/FLP MXene composites with weight ratios of 1:1 (red), 1:2 (green), 1:5 (blue), and 1:10 (cyan).

The FT-IR spectra of FLP MXene, PTh, and PTh/FLP MXene composites with varying ratios are displayed in Figure 4. Here, FLP MXene was prepared with 20% HF solution. Various functional groups, such as O–Ti–O, –OH, C–F, and C=O, are observed for the FLP MXene sample (Figure 4a) [25]. The FT-IR spectra of the other FLP MXenes that were treated with different concentrations of HF solutions appear similar, as presented in Figure S3. Characteristic absorption peaks are also found for PTh nanospheres (Figure 4b). A small peak at 690 cm^−1^ is assigned to the C–S stretching vibration, and two peaks appearing at 1220 and 2920 cm^−1^ correspond to C–H deformation vibration and stretching vibration modes of the C–H bond. A noisy peak around 1460 cm^−1^ originates from the C=C stretching vibration [26]. Figure 4c shows the FT-IR spectra of PTh/FLP MXene composites. They reveal characteristic peaks of both PTh nanospheres and FLP MXene at wavenumbers close to those in their pristine states. For the ease of figuring out the curves, the peaks belonging to PTh nanospheres are notated in black, while the other peaks coming from FLP MXene are marked in red. Interestingly, the FT-IR spectrum of a composite with equal fractions of PTh and MXene resembles that of pure PTh nanospheres, but the similarity and clarity of the spectrum becomes weaker as the fraction of MXene relative to PTh increases. Furthermore, the location of C–S peak shifts toward a lower wavenumber as compared to pure PTh nanospheres, which may be attributed to weak hydrogen bonding formation between S of the thiophene ring and the –OH group on the FLP MXene surface, enabling good adherence of PTh nanospheres to the MXene surface. All of these results indicate the successful synthesis of PTh/FLP MXene composites.

### 2.3. Optical Characteristics

**Figure 5:**
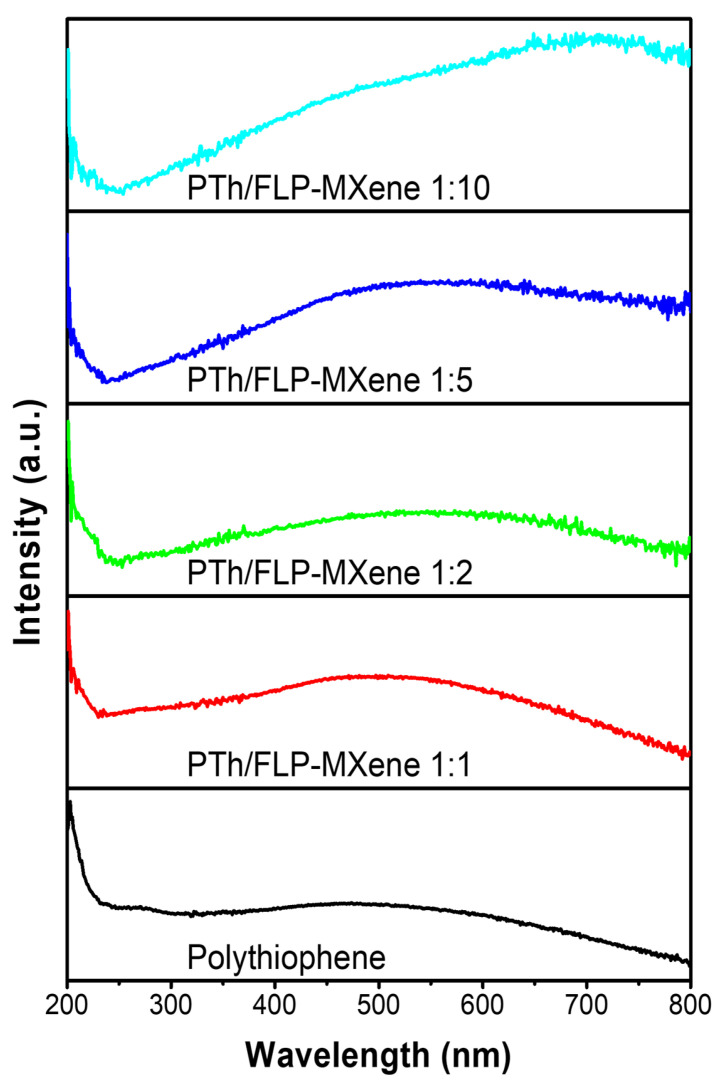
UV-Vis absorption spectra of PTh nanospheres (black) and PTh/FLP MXene composites with weight ratios of 1:1 (red), 1:2 (green), 1:5 (blue), and 1:10 (cyan).

To understand the optical characteristics of samples, UV-Vis absorption spectroscopy was first utilized. Figure 5 shows the UV-Vis absorption spectra of PTh nanospheres and PTh/FLP MXene nanocomposites of different weight fractions. For comparison, the absorption spectra of control MXene and various FLP MXenes are provided in Figure S4, and they appear like straight lines without meaningful curvatures. The PTh nanospheres exhibit a broad curve slowly ascending as the wavelength decreases. The curve in general spreads over the visible light to ultraviolet range, and it centers at approximately 470 nm. Even when the PTh nanospheres are hybridized with FLP MXene, the overall curve shape does not change much. However, the curve sharpens, and its center gradually moves to higher wavelength as the relative weight fraction of FLP MXene increases. For instance, the curve centers of PTh/FLP MXene composites with PTh:MXene = 1:1, 1:2, and 1:10 located at about 500, 560, and 710 nm, respectively. This means that the response of the composite to light intensifies, and its core spectral part shows a red shift as compared to pure PTh nanospheres. This modification of optical characteristics is desirable for a photocatalyst since most solar spectrum falls in the visible light range. Nevertheless, the composites with MXene weight fractions exceeding 2 appear to lose their optical advantage, as the red shift becomes excessive in those samples.

**Figure 6:**
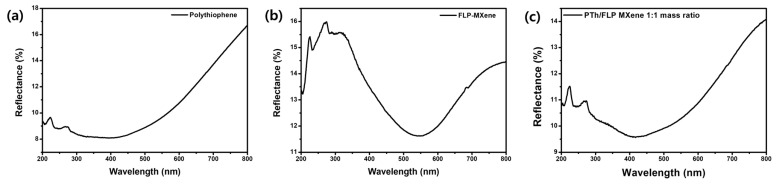
DRS spectra of (**a**) PTh nanospheres, (**b**) FLP MXene, and (**c**) PTh/FLP MXene composite (1:1).

To analyze the optical absorption characteristics of PTh nanospheres, FLP MXene and PTh/FLP MXene composite more exquisitely, DRS measurements were performed. As can be seen in Figure 6a, pure PTh nanospheres show a broad light absorption curve spanning over visible light to ultraviolet, the center of which places at ~400 nm. This observation generally agrees with the result from UV-Vis absorption spectroscopy. On the other hand, the FLP MXene reveals a rather sharp absorption curve in the visible light range, as shown in Figure 6b. It centers at 548 nm, which is far larger than that of PTh nanospheres. As displayed in Figure 6c, the PTh/FLP MXene composite exhibits a DRS curve that compromises DRS spectra of both PTh nanospheres and FLP MXene. Both the sharpness and central position of its DRS curve are located in between those of PTh nanospheres and FLP MXene. To estimate the band gaps of the materials, we made Kubelka–Munk plots using these DRS spectra. As shown in Figure S5, the band gaps of PTh nanospheres, FLP MXene, and PTh/FLP MXene composite are estimated to be 2.45, 1.45, and 1.14 eV, respectively, which moderately agree with the previous reports [27,28,29,30,31]. These results implicate that the light absorption characteristics of the composite can be elaborately tuned to harvest the optimal range of light.

### 2.4. BET Analysis

Two important features for adsorption are total surface area and an electrostatic interaction between adsorbent and adsorbate. To analyze the surface area and porosity of samples, BET N_2_ adsorption-desorption tests were conducted. Figure S6 shows the adsorption–desorption isotherms of FLP MXene, PTh nanospheres, and the PTh/FLP MXene composite (1:1). They all fall in the type IV category defined by the IUPAC. From those, BET surface areas were calculated and are provided in Table 1. Furthermore, pore size distributions and pore volumes were analyzed by employing the Barret, Joyner, and Halenda (BJH) method [32,33]. The results are given in Figure S6, and the calculated pore volume and average pore size are summarized in Table 1. The surface area of the composite (32.01 m^2^/g) is the largest, which is marginally larger than that of PTh nanospheres (29.05 m^2^/g). The ample surface areas of PTh nanospheres and the PTh/FLP MXene composite can make MB molecules easily adsorb on the surfaces. In addition, it was reported that the surface of PTh powder is negatively charged, proven by a negative zeta potential [34]. Because MB is a cationic dye, a strong electrostatic attraction between PTh nanospheres and MB molecules are highly probable, which facilitates MB adsorption on the surface of PTh nanospheres or the PTh/FLP MXene composite.

### 2.5. Photodegradation and Adsorption of Organic Dyes

**Figure 7:**
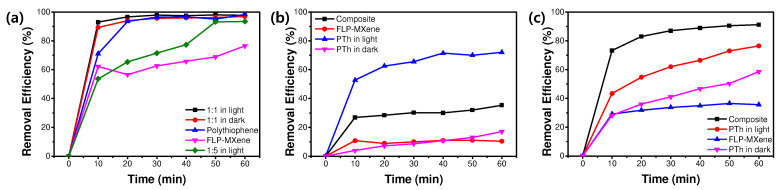
Removal efficiencies of (**a**) MB, (**b**) OG, and (**c**) RhB.

Dye removal tests were conducted using the aforementioned materials as photocatalysts. At first, the tests were carried out for MB solution, which served as a basic dye solution. Figure S8a–d show UV-Vis absorption spectra of MB solutions containing different sorts of photocatalysts. Those absorption spectra arise from MB molecules in the solutions, and the intensity (absorbance) of a primary peak appearing at 664 nm obviously decreases with irradiation time for all solutions. However, the degree of absorbance reduction of MB solution with the PTh/FLP MXene composite (1:1) overwhelms those of PTh nanospheres, FLP MXene, and another PTh/FLP MXene composite (1:5). This indicates the superior MB-removing capability of the composite with the weight ratio of 1 to 1, which is also supported by rapid discoloration of MB solution, as shown in Figure S7. Surprisingly, most absorbance reduction occurs within the initial 10 min of irradiation for this composite. This is too short to be ascribed only to photodegradation of MB molecules. Indeed, almost the same magnitude of absorbance reduction is observed in dark conditions (Figure S8e), which means that the MB removal is not caused by the photodegradation mechanism, but probably by MB adsorption onto the composite surface. The dye removal efficiencies were calculated by

(1) $$Removal\;efficiency\left(\%\right)=\frac{C_{0}-C_{t}}{C_{0}}\times 100$$

here, *C*_0_ and *C_t_* represent the concentrations of a dye at the time of 0 and t, and these concentrations are estimated from the relative absorbance of a characteristic peak at each time. The material-dependent MB removal efficiencies are plotted in Figure 7a. It is obvious that the PTh/FLP MXene composite (1:1) is the best material, and the MB-removing event occurs most actively within an initial 10 min. This composite exhibits the very high removal efficiencies of 93.1% and 97.8% in 10 min and 1 h, respectively, under light. These efficiencies are much better than those of conventional photocatalysts, such as TiO_2_, ZnO, and g-C_3_N_4_ [35,36,37,38]. The MB removal efficiency of the composite does not exhibit a significant difference in light and dark conditions, and it shows only a slight advantage over pure PTh nanospheres. As mentioned above, these are most likely attributed to the adsorption of MB molecules onto the adsorbent surface, and PTh nanospheres may be the major component responsible for the adsorption.

The OG and RhB removal efficiencies of the photocatalysts were further evaluated. Figure S9a–c display the time-dependent UV-Vis absorption spectra of OG solutions including different photocatalysts in light or dark conditions. The characteristic peak of OG at 484 nm gradually shrinks with time, but the intensity reduction is much smaller than the case for MB solution. The intensity of the PTh/MXene composite reduces less than PTh nanospheres at the same light illumination time, and the PTh nanospheres show only a slight intensity decrease in dark conditions. These result in time-dependent OG removal efficiencies of different photocatalysts, as provided in Figure 7b. The small efficiency of PTh nanospheres in the dark (<20%) is ascribed to the electrostatic repulsion between the PTh surface and OG molecules, which are anionic. The OG removal efficiency of PTh nanospheres jumps up to 72% in 1 h of light illumination, representing relatively good electron (e)-hole (h) pair generation, followed by photodegradation of OG under light. It is speculated that a significant drop in the OG removal efficiency of PTh/MXene composite results from a PTh surface shadowing effect by FLP MXene flakes. A considerable surface of PTh nanospheres that are a major light harvester may be blocked by MXene, leading to a degradation in their photocatalytic activity [39].

Figure S9d–f show the similar test results for RhB solutions. For RhB, the dye removal efficiency of PTh nanospheres in dark falls in between the values for MB and OG (Figure 7c). For example, it reaches 58.6% after 1 h in dark conditions. This moderate removal efficiency is likely to relate to almost neutral charge characteristics of RhB. In this case, RhB molecules may adsorb on the material surface by van der Waals interaction. The RhB removal efficiency increases to 76.5% when the solution is irradiated for the same period of time. The best performance comes from the PTh/FLP MXene composite, which degrades 91.3% of RhB after irradiated for 1 h. This significant enhancement in RhB removal efficiency is attributed to synergistic works of PTh nanospheres and FLP MXene, which will be discussed later. As presented in Figure S10, the sole FLP MXene is not effective in removing both OG and RhB.

### 2.6. Mechanisms for Dye Removal

The outstanding MB removal efficiencies of PTh nanospheres and PTh/FLP MXene composite result from the active surface adsorption of MB molecules caused by a strong electrostatic attraction between the adsorbent and MB. Moreover, their large surface areas facilitate the adsorption process. Likewise, the poor OG removal efficiency of PTh nanospheres in the dark is attributed to an electrostatic repulsion between PTh and OG. Despite this barrier, the increment of removal efficiency by 55% under light advocates the vital photoactivity of PTh nanospheres. In terms of surface adsorption, RhB falls in the medium range due to its neutral charge characteristics.

To further analyze the dye adsorption kinetics of the PTh/FLP MXene composite (1:1), the pseudo-first-order and pseudo-second-order kinetic models were comparatively employed. The two models are formulated as follows [40,41,42]:

(2) $$\ln C=\ln C_{0}-\;K_{1}t\;\;(\mathrm{pseudo-first-order})$$

(3) $$\frac{1}{C}=\;\frac{1}{C_{0}}+\;K_{2}t\;\;(\mathrm{pseudo-second-order})$$

**Figure 8:**
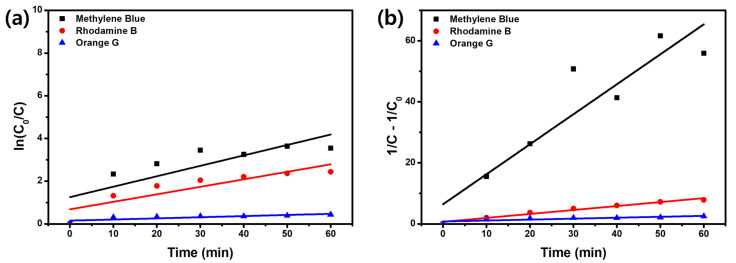
(**a**) Pseudo-first order kinetic and (**b**) pseudo-second order kinetic models for MB (black), OG (blue), and RhB (red). Here, PTh/FLP MXene composite (1:1) was used as an adsorbent.

Here, *C*_0_ is the initial dye concentration, *C* is the concentration at time t, and *K*_1_ and *K*_2_ are the rate constants. The pseudo-first-order model can be reduced to the Langmuir-Hinshelwood model. Two graphs are constructed in Figure 8a,b, based on the respective models. While linear fitting is applicable to both graphs, the fitting to the pseudo-second-order kinetic model appears to be more reliable. From the linear fitting, the rate constants and R^2^ values were estimated, and they are summarized in Table 2. For all types of dyes, the R^2^ values of the pseudo-second-order model are higher, suggesting that the model is more adequate for this work. Notably, the rate constant for MB overpowers those for the other dyes. This undoubtedly represents that the surface adsorption of MB molecules is the key removal mechanism for MB.

**Figure 9:**
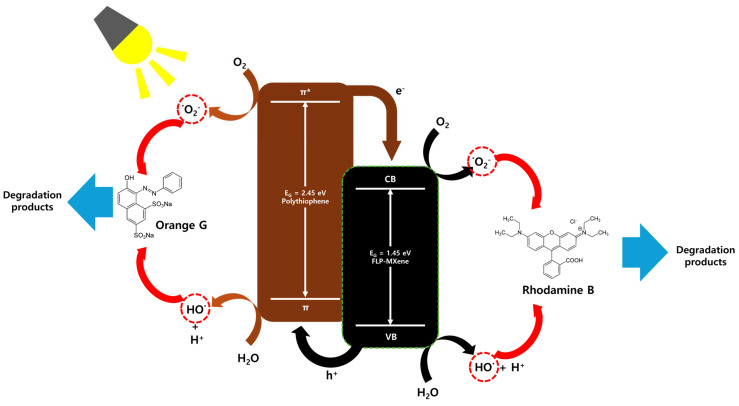
Schematic mechanisms for photodegradation of organic dyes activated by the PTh/FLP MXene composite.

For OG and RhB, the direct adsorption of molecular species onto the surface is rather weak. Thus, photodegradation plays as the main dye-removing mechanism for these dye solutions, as schematically illustrated in Figure 9. PTh nanospheres are the core functional component for OG photodegradation, with no contribution from FLP MXene. In this case, PTh nanospheres efficiently absorb the photon energy, leading to the generation of a substantial number of electron-hole (e–h) pairs. Negatively charged electrons and positively charged holes react with oxygen (O_2_) and H_2_O molecules, which subsequently generate highly active superoxide anions (∙O_2_^−^) and hydroxyl radicals (·OH), as described in the following reactions.

(4) $$Polythiophene+hv\;\to Polythiophene+(e^{-}+\;h^{+})$$

(5) $$e^{-}+\;O_{2}\;\to \;\;\cdot O_{2}^{-}$$

(6) $$h^{+}+\;H_{2}O\to \cdot OH+H^{+}$$

(7) $$\cdot O_{2}^{-}\;\;or\;\;\cdot OH+\;dye\;\to \;\;\;Degradation\;products$$

These active radicals are responsible for photodegradation of OG molecules. As for RhB, the photodegradation mechanism works more effectively by a collaboration of PTh nanospheres and FLP MXene, as seen in Figure 9. Now, the e–h pairs produced mostly by PTh nanospheres are well-separated by the coupled FLP MXene. Due to the elaborate band alignment of two structural components, electrons move from PTh to MXene, while holes migrate in the reverse direction. Those migrated electrons and holes can stay for a longer time in FLP MXene and PTh nanospheres, respectively, initiating the RhB photodegradation mechanism more actively. This is why the RhB removal efficiency of PTh/FLP MXene composite dominates over those of pure PTh nanospheres and FLP MXene.

The surface adsorption mechanism was previously supported by the high removal efficiency in dark conditions and the high adsorption rate constant. This is particularly true for MB. However, the removal efficiencies can be greatly elevated under light for the other dyes. To better understand the photoactivity of the PTh/FLP MXene composite (1:1), the photocurrent response was measured using a Keithley 2450 Source meter at a source voltage of 0 V. As presented in Figure S11, the current level obviously increases when the light is on, and it returns to the initial level when the light is off. The current increase under light illumination represents the photocurrent, which is attributed to photoexcited electron-hole pair generation. These photogenerated carriers can actively participate in the formation of active radicals, as explained above.

### 2.7. Recyclability, Radical Scavenging, and Expandible Degradation Tests

**Figure 10:**
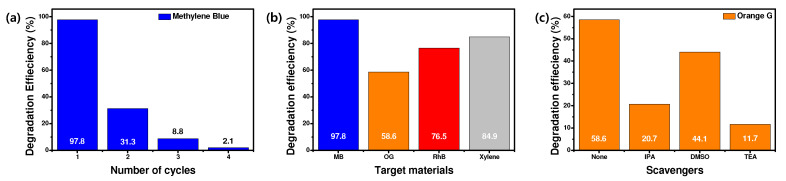
(**a**) Cycle-dependent MB degradation efficiencies. (**b**) Degradation efficiencies of various organic dyes and pollutants. (**c**) Free radical scavenging test results using three different scavengers.

The recyclability of the PTh/FLP MXene composite (1:1) was further evaluated, using MB as a target dye. For it, the composite was collected after a test at each cycle and cleaned for 20 min using DI water. The cycle-dependent degradation efficiency of MB is presented in Figure 10a. An identical elapse time of 60 min was applied to all cycles. The first-cycle degradation efficiency records 97.8%. However, a sharp decline in efficiency is observed from the second cycle. This is because the MB is removed by a surface adsorption mechanism and the electrostatic attraction between the PTh nanospheres and MB molecules is too strong to separate the adsorbed MB from the surface. This indicates that the composite cannot be directly recycled for repeated use. However, the PTh nanospheres can be detached from the FLP MXene surface by a strong sonication, and new PTh nanospheres can be easily attached to the MXene surface.

In addition, an additional degradation test was conducted using a new organic pollutant to diagnose the expandability of the composite. Xylene was selected as a target pollutant, and a 12.5 μmol xylene solution of 40 mL in volume was treated with 20 mg of the PTh/FLP MXene composite (1:1). As shown in Figure 10b, xylene exhibits a degradation efficiency of 84.9%, which is higher than those of other organic dyes, except for MB. This suggests that our composite could be widely applied for removing diverse organic pollutants in wastewater.

A free radical scavenging test was carried out, using OG as a target dye and the PTh/FLP MXene composite (1:1) as a photocatalyst. The test condition was basically the same as the dye degradation tests previously described. For this test, three different scavengers, i.e., DMSO, TEA, and IPA were used, which target active superoxide anion radicals (∙O_2_^−^), hydroxyl radicals (·OH), and holes (h^+^), respectively. Each scavenger was introduced into the solution at a concentration of 1 mmol/L, and OG degradation efficiency was measured after 60 min of light illumination. As presented in Figure 10c, the results reveal that both types of radicals and holes are present in irradiated OG solution. Particularly, holes and hole-induced hydroxyl radicals are major species that degrade OG by photocatalytic reaction.

## 3. Materials and Methods

### 3.1. Materials and Reagents

Ti_3_AlC_2_ powder (400 mesh) was purchased from Nanochemazone (Leduc, Alberta, Canada). Hydrofluoric acid (HF, 48–51%) and dimethyl sulfoxide (DMSO, ACS reagent, 99.9%) were supplied by Thermo Scientific (Fair Lawn, NJ, USA). Methanol (≥99.9%), ammonium persulfate (APS, ACS reagent, ≥98.0%), isopropyl alcohol (IPA), triethanolamine (TEA, reagent grade, 98%), thiophene (≥99%), methylene blue (MB), orange G (OG), and rhodamine B (RhB) were all obtained from Sigma-Aldrich Korea (Seoul, Republic of Korea). Cetyltrimethylammonium bromide (CTAB, 99%) was purchased from Daejung Chem (Siheung, Republic of Korea).

### 3.2. Synthesis of Standard Ti_3_C_2_T_X_ MXene

**Figure 11:**
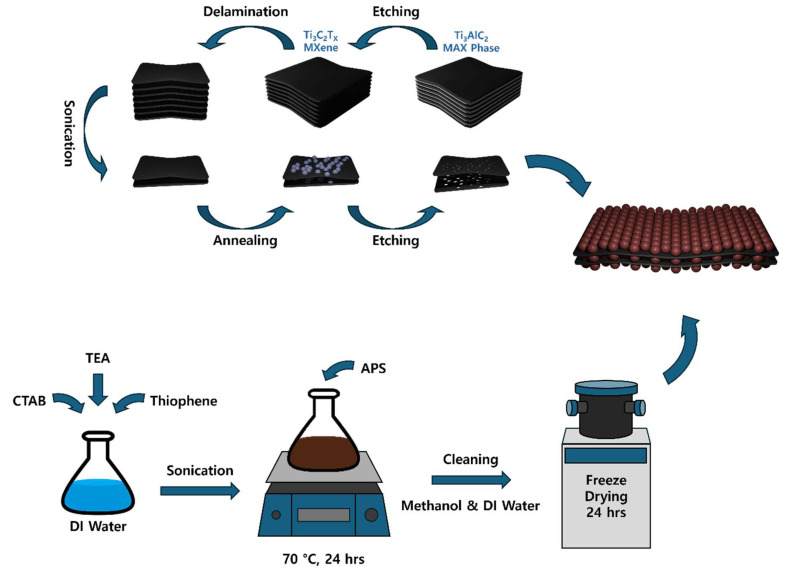
Schematic procedures for the synthesis of the PTh/FLP MXene composite.

Standard Ti_3_C_2_T_x_ MXene was synthesized from Ti_3_AlC_2_ MAX phase by selectively etching out the Al layers, as depicted schematically in Figure 11. Briefly, a 5 g of Ti_3_AlC_2_ powder was gradually added to 160 mL of HF solution (~50%), followed by magnetic stirring at 500 rpm for 24 h at 50 °C to fully remove the Al layers. After etching, the resulting product was collected via centrifugation at 8000 rpm, then vacuum-filtered. De-ionized (DI) water was continuously poured during filtration to neutralize the acidity until a pH of ~7 was reached. The filtered product was freeze-dried for 12 h, then further dried in a vacuum oven for 24 h at 40 °C to remove residual moisture. Finally, the MXene was ground into a fine powder for approximately 20 min using a mortar and pestle. This multilayer Ti_3_C_2_T_x_ MXene served as a control sample.

### 3.3. Synthesis of Few Layered Porous Ti_3_C_2_T_X_ MXene

To prepare few-layered porous (FLP) Ti_3_C_2_T_x_ MXene, a 500 mg of standard Ti_3_C_2_T_x_ MXene was mixed with 20 mL of DMSO and stirred at 300 rpm for 24 h to facilitate intercalation. The mixture was then washed with DI water to remove any excess DMSO and refrigerated for 12 h. Right after refrigeration, the product was freeze-dried for 24 h. To enhance porosity, heat-induced TiO_2_ particle formation was used, followed by the selective etching of the particles. In more detail, the FLP MXene sample was heated in a muffle furnace for 2 h at 200 °C and then etched for 8 h in 20% HF solution. Here, a subtle tuning in HF concentration and etching time was made to find an optimal pore distribution. Finally, the product was thoroughly washed with DI water to remove residual HF, and dried in a vacuum oven for 24 h at 40 °C. These processes are schematically presented in the upper part of Figure 11.

### 3.4. Synthesis of Polythiophene

Polythiophene was synthesized via chemical oxidative polymerization, using CTAB and triethanolamine as the surfactant and stabilizer, respectively. For this, two kinds of solutions were independently prepared at first. To ensure micelle formation (solution 1), 2.06 g of CTAB, 5.27 g of triethanolamine, and 2.5 mL of thiophene were dissolved in 30 mL of DI water and sonicated for 30 min. Concurrently, 8.26 g of ammonium persulfate was dissolved in 20 mL of DI water as an oxidizing agent (solution 2). Then, solution 1 was placed in a 70 °C oil bath with stirring at 300 rpm, and solution 2 was added dropwise to solution 1 over 24 h, which induced the polymerization of thiophene into polythiophene in a controllable manner. After the reaction, the polythiophene was washed with DI water and methanol, stored in a refrigerator for 12 h, and freeze-dried for 24 h. The key steps for PTh synthesis are drawn in the bottom part of Figure 11.

### 3.5. Synthesis of Composites

To synthesize PTh/FLP MXene composites, a facile solution hybridization technique was employed. To this aim, pre-synthesized FLP Ti_3_C_2_T_x_ MXene flakes and PTh nanospheres were simply mixed in 30 mL of DI water and stirred at 300 rpm for 3 h at 40 °C. The weight ratios between FLP MXene and PTh were controlled in the range of 1:1 to 1:5 to examine the effect of their relative fractions. The resulting composite was collected by centrifugation at 10,000 rpm and vacuum-dried for 24 h at 40 °C.

### 3.6. Material Characterizations

The layer structures, surface morphologies, and compositions of control MXene, FLP MXene, PTh, and PTh/FLP MXene composites were analyzed by a field emission scanning electron microscope (FE-SEM, Hitachi SU 8600, Tokyo, Japan) equipped with an energy-dispersive X-ray (EDX) spectrometer. The crystalline characteristics of control and FLP MXenes were examined by X-ray diffraction (XRD, Rigaku, Smartlab, Tokyo, Japan) with copper (Cu) Kα radiation. The acceleration voltage and electron beam current of the X-ray generator were set at 40 kV and 30 mA, respectively. The major functional groups and their molecular motions were monitored using Fourier-transform infrared spectroscopy (FT-IR, JASCO FT/IR-4600, Tokyo, Japan). The optical absorption spectra and diffuse reflectance spectra (DRS) of samples were measured by a UV-visible spectrophotometer (UV-Vis, Cary 50 Bio, Santa Clara, CA, USA) and diffuse reflectance spectroscopy (JASCO V-770, Tokyo, Japan), respectively. To investigate the photoactivity of the PTh/FLP MXene composite (1:1), the photocurrent response was measured using a Keithley 2450 Source meter at a source voltage of 0 V. Furthermore, the Brunauer–Emmett–Teller (BET) surface areas and average pore sizes of selected samples were analyzed by a nitrogen (N_2_) adsorption–desorption apparatus (Micromeritics, ASAP 2020, Norcross, GA, USA).

### 3.7. Photocatalytic and Physical Adsorption Dye Degradation Tests

Three types of organic dyes (MB, RhB, OG) were used for photocatalytic and physical adsorption tests. For that, individual aqueous dye solutions were first prepared with a fixed concentration of 12.5 μM. Of the three, MB was used as a basic dye to estimate the photocatalytic and adsorptive activities of various materials, like FLP Ti_3_C_2_T_x_ MXene, PTh, and PTh/FLP MXene composites. A 20 mg of photocatalyst was added to 40 mL of 12.5 μM MB solution that was contained in a 50 mL glass vial. The tests were conducted at room temperature and pH of 6~7. The MB solutions including different photocatalysts were irradiated by the stimulated light that was generated by a solar simulator (Asahi spectra, HAL-320, Torrance, CA, USA) under constant stirring (300 rpm). The absorbance of a characteristic peak corresponding to MB was monitored with time over a span of 60 min. The best photocatalyst was singled out from these serial tests, and it was directly applied to the other organic dyes (RhB, OG) to evaluate its removal efficiencies.

## 4. Conclusions

In conclusion, we successfully synthesized PTh nanospheres/FLP MXene composites by a simple sequential solution method. The FLP MXene was prepared from multilayer Ti_3_C_2_T_x_ MXene via DMSO intercalation, heat-induced oxidation, and selective removal of TiO_2_ nanoparticles. In parallel, PTh nanospheres were synthesized by chemical oxidative polymerization. An optimized composite with the equal weight fractions of MXene and PTh showed the uniform, dense distribution of small PTh nanospheres on the highly crystalline FLP MXene, leading to an enhanced surface area. Moreover, the PTh/FLP MXene composite exhibited a broad light absorption peak over a range of visible light to ultraviolet, revealing its improved light-harvesting capability. The composite showed superior dye removal efficiencies for multiple organic dyes such as methylene blue (MB), rhodamine B (RhB), and orange G (OG), when it was employed as a photocatalyst. In particular, 93.1% of MB was removed from MB solution within an initial 10 min of light illumination. It was disclosed that both adsorption and photodegradation work collaboratively for the dye removal. The detailed dye removal mechanisms were discussed on the basis of electrostatic interaction and effective charge separation process. The promising results of this work suggest that the PTh/FLP MXene composite could serve as an effective and sustainable solution for addressing aqueous pollution problem caused by organic dyes.

## Figures and Tables

**Table 1:** BET surface areas, pore volumes, and pore sizes of PTh nanospheres, FLP MXene, and the PTh/FLP MXene composite (1:1).

Photocatalyst	BET Surface Area (m^2^/g)	Pore Volume (cm^3^/g)	Pore Size (nm)
Polythiophene	29.05	0.147	20.19
FLP MXene	9.73	0.0321	13.18
PTh/FLP MXene composite	32.01	0.0616	7.70

**Table 2:** Parameters obtained from dye removal kinetic models.

Organic Dye	Pseudo-First Order		Pseudo-Second Order	
	K_1_	R^2^	K_2_	R^2^
MB	0.048	0.61	0.98	0.84
RhB	0.035	0.74	0.13	0.97
OG	0.0053	0.56	0.03	0.61

## Data Availability

The data presented in this study are available on request from the corresponding author.

## Supplementary Materials

The following supporting information can be downloaded at: https://www.mdpi.com/article/10.3390/molecules30061393/s1, Figure S1. SEM images of PTh nanospheres: (a) low magnification, (b) higher magnification; Figure S2. SEM images of PTh/FLP MXene composites depending on the relative weight (mass) ratio of FLP MXene to PTh. (a,b) PTh:MXene = 1:1, (c) PTh:MXene = 1:5, (d) PTh:MXene = 1:10; Figure S3. FT-IR spectra of control MXene, few-layered MXene, and FLP MXenes that were prepared with different concentrations of HF solutions; Figure S4. UV-Vis absorption spectra of control MXene, few-layered MXene, and FLP MXenes that were prepared with different concentrations of HF solutions; Figure S5. Kubelka–Munk plots of (a) PTh nanospheres, (b) FLP MXene, and (c) PTh/FLP MXene composite (1:1). The band gap energies of the materials were estimated from the respective plots; Figure S6. The N_2_ adsorption-desorption isotherms of (a) FLP MXene, (b) PTh nanospheres, and (c) PTh/FLP MXene composite (1:1). Differential pore volumes vs. pore size curves of (d) FLP MXene, (e) PTh nanospheres, and (f) PTh/FLP MXene composite (1:1); Figure S7. Photo images showing (a) initial MB solution and (b) its rapid discoloration with irradiation time; Figure S8. Time-dependent UV-Vis absorption spectra of MB solutions containing (a) FLP MXene, (b) PTh nanospheres, (c) PTh/FLP MXene composite (1:1), and (d) PTh/FLP MXene composite (1:5). All tests were performed under light. (e) Time-dependent UV-Vis absorption spectra for PTh/FLP MXene composite (1:1) in dark.; Figure S9. (a–c) Time-dependent UV-Vis absorption spectra of OG solutions containing PTh/FLP MXene composite (1:1) and PTh nanospheres in light or dark conditions. (d–f) Time-dependent UV-Vis absorption spectra of RhB solutions containing PTh/FLP MXene composite (1:1) and PTh nanospheres in light or dark conditions. For (a,d), PTh/FLP MXene composite (1:1) was used while PTh nanospheres were used for the others; Figure S10. Time-dependent UV-Vis absorption spectra of (a) OG and (b) RhB solutions. Here, FLP MXene was used as a photocatalyst; Figure S11. Photocurrent response of PTh/FLP MXene composite (1:1).
