# Supplementary material for: Polythiophene/Ti3C2TX MXene Composites for Effective Removal of Diverse Organic Dyes via Complementary Activity of Adsorption and Photodegradation
Source: Molecules. 2025 Mar 20;30(6):1393. doi: 10.3390/molecules30061393 (PMC11944630; doi:10.3390/molecules30061393)
Supplement: Supplementary file 1 [file molecules-30-01393-s001.zip › molecules-3508482-supplementary.pdf]

Supporting Information for

Polythiophene/$Ti_3C_2T_X$ MXene Composites for Effective Removal of Diverse Organic Dyes via Complementary Activity of Adsorption and Photodegradation

Young-Hwan Bae[a], Seongin Hong[a,*], Jin-Seo Noh[b,*]

[a]*Department of Semiconductor Engineering, Gachon University, 1342 Seongnam-daero, Sujeong-gu, Seongnam-si, Gyeonggi-do 13120, Korea*

[b]*Department of Physics, Gachon University, 1342 Seongnam-daero, Sujeong-gu, Seongnam-si, Gyeonggi-do 13120, Korea*

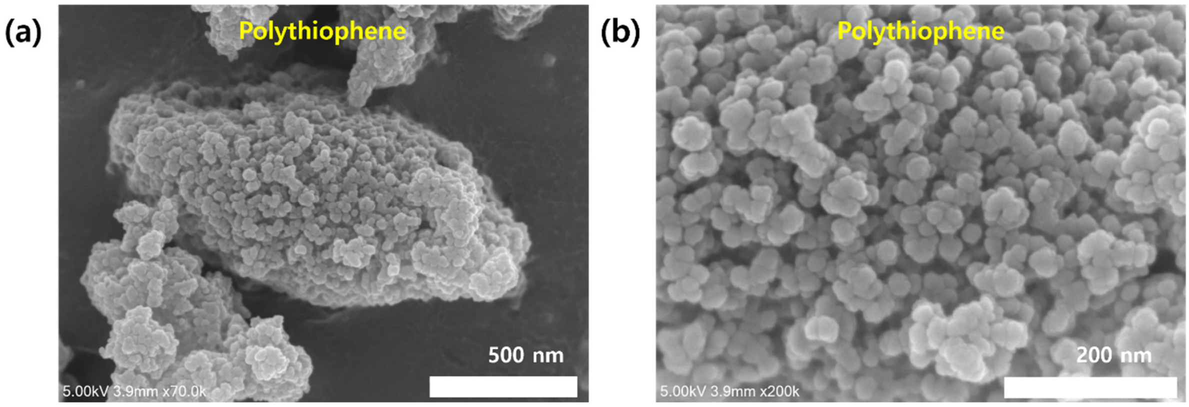

Figure S1. SEM images of PTh nanospheres: (a) low magnification, (b) higher magnification.

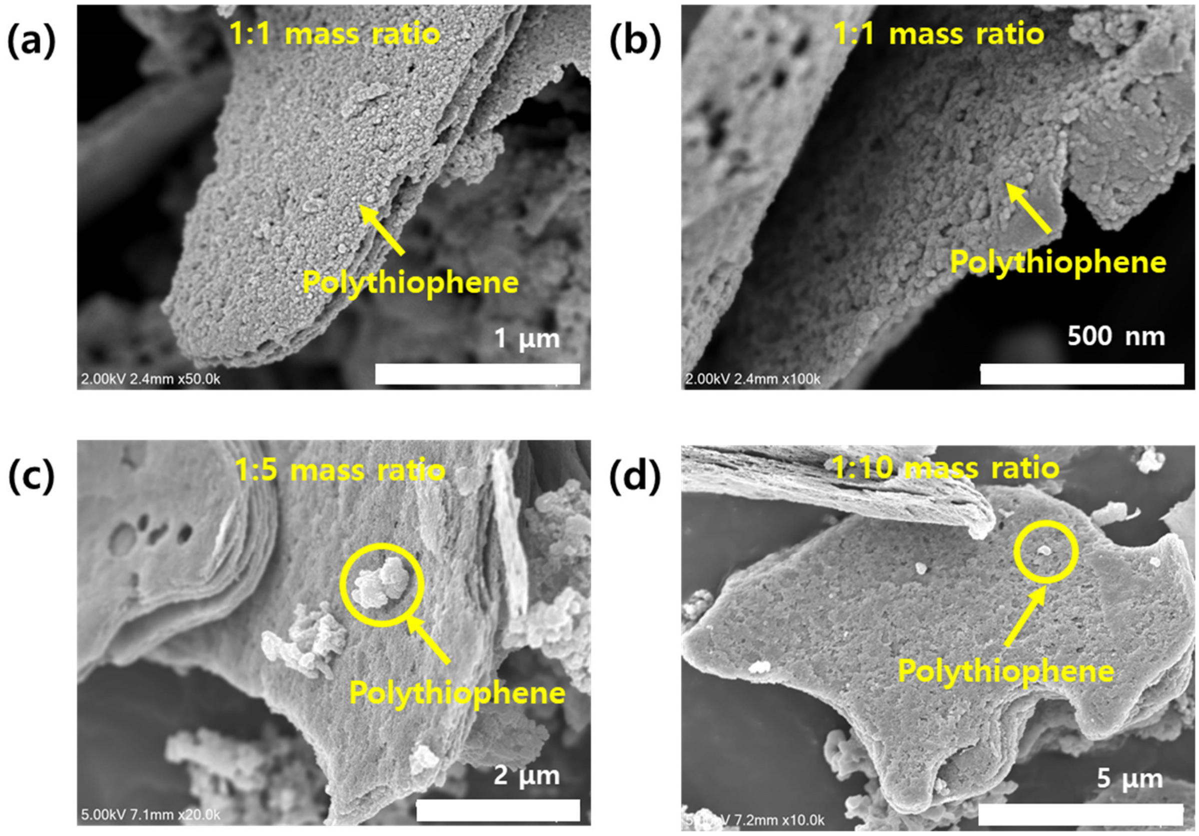

Figure S2. SEM images of PTh/FLP MXene composites depending on the relative weight (mass) ratio of FLP MXene to PTh. (a, b) PTh:MXene=1:1, (c) PTh:MXene=1:5, (d) PTh:MXene=1:10.

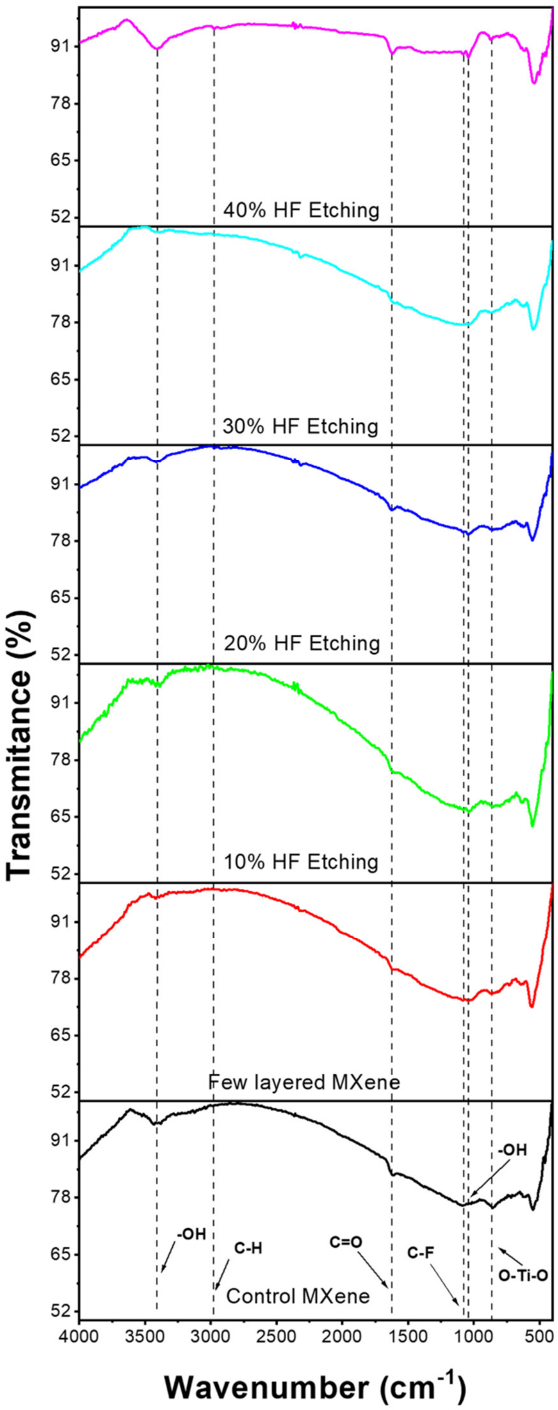

Figure S3. FT-IR spectra of control MXene, few-layered MXene, and FLP MXenes that were prepared with different concentrations of HF solutions.

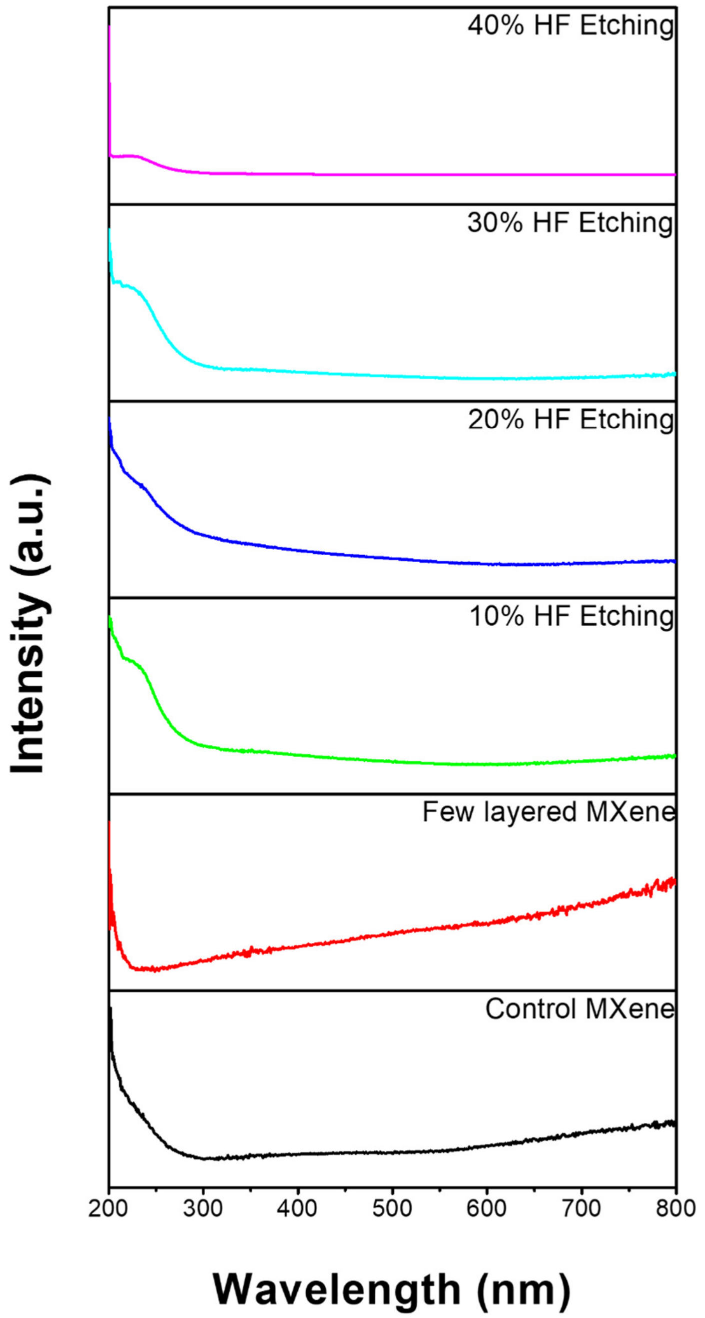

Figure S4. UV-Vis absorption spectra of control MXene, few-layered MXene, and FLP MXenes that were prepared with different concentrations of HF solutions.

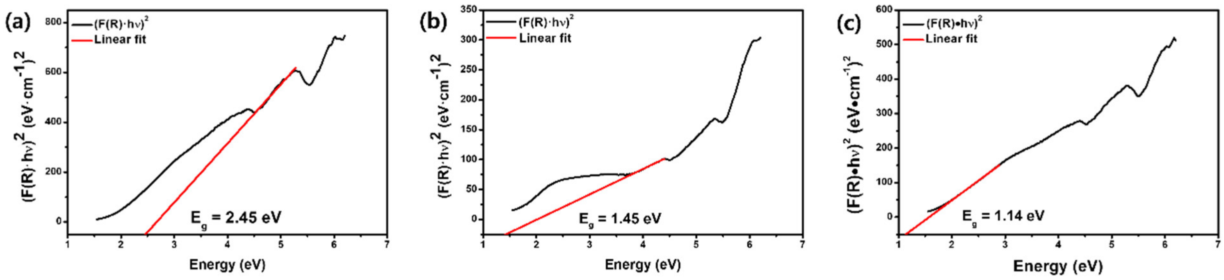

Figure S5. Kubelka-Munk plots of (a) PTh nanospheres, (b) FLP MXene, and (c) PTh/FLP MXene composite (1:1). The band gap energies of the materials were estimated from the respective plots.

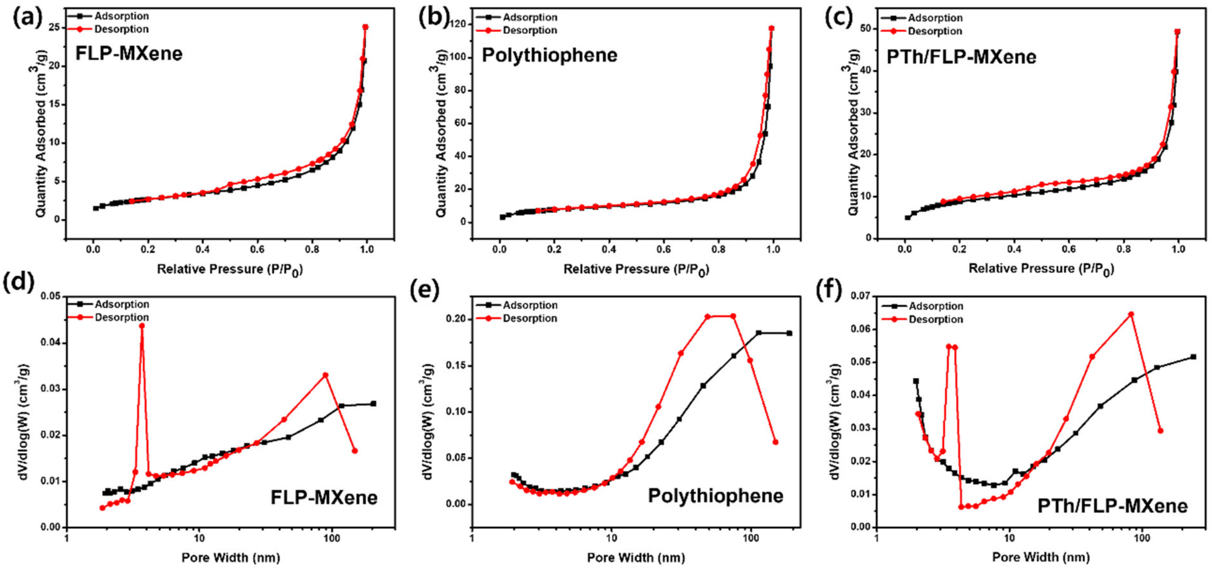

Figure S6. The N_2 adsorption-desorption isotherms of (a) FLP MXene, (b) PTh nanospheres, and (c) PTh/FLP MXene composite (1:1). Differential pore volumes vs. pore size curves of (d) FLP MXene, (e) PTh nanospheres, and (f) PTh/FLP MXene composite (1:1).

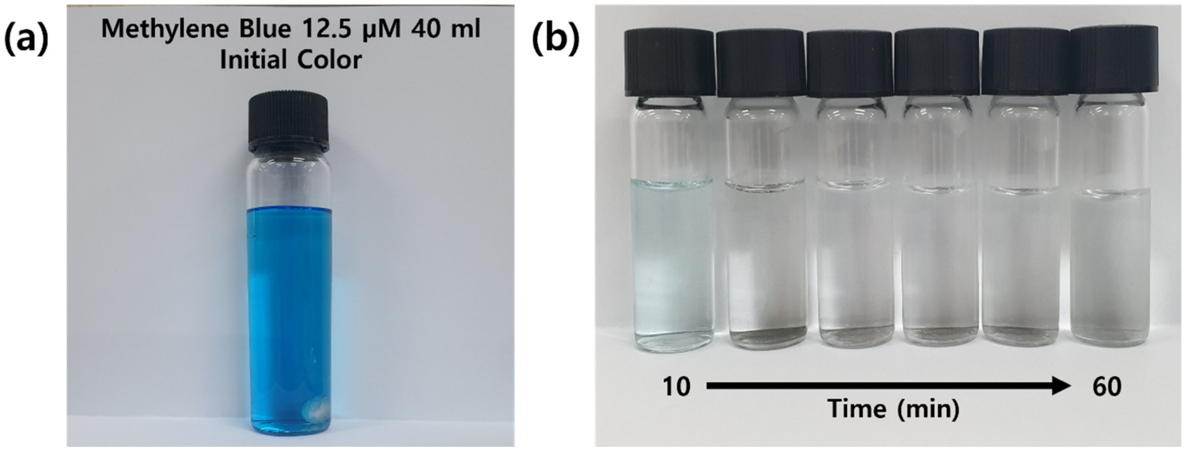

Figure S7. Photo images showing (a) initial MB solution and (b) its rapid discoloration with irradiation time.

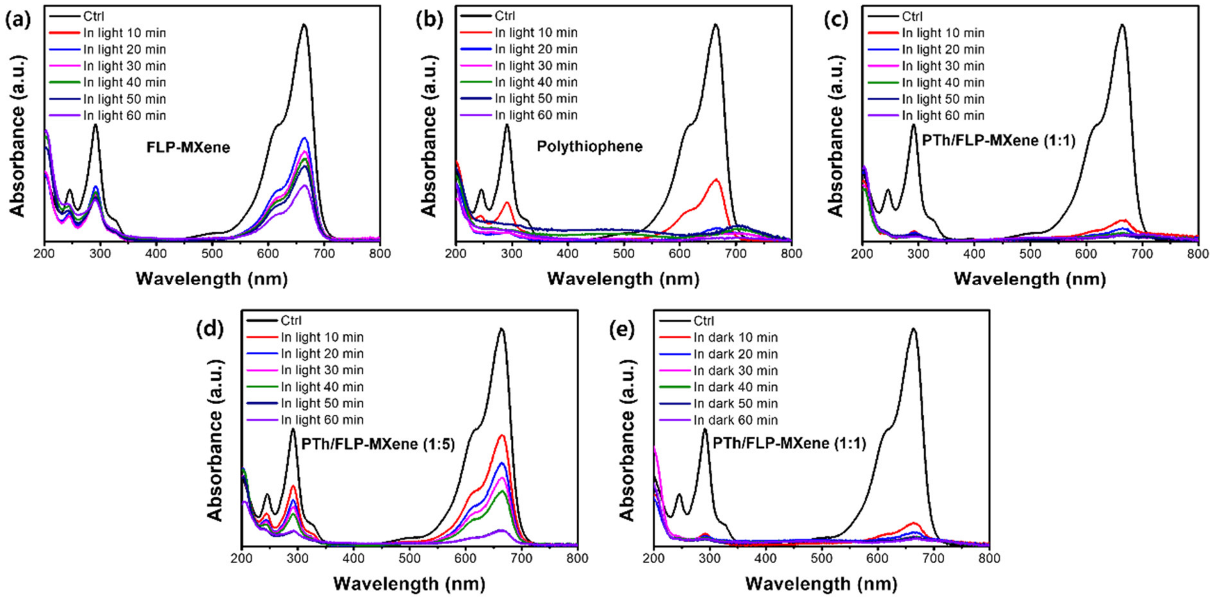

Figure S8. Time-dependent UV-Vis absorption spectra of MB solutions containing (a) FLP MXene, (b) PTh nanospheres, (c) PTh/FLP MXene composite (1:1), and (d) PTh/FLP MXene composite (1:5). All tests were performed under light. (e) Time-dependent UV-Vis absorption spectra for PTh/FLP MXene composite (1:1) in dark.

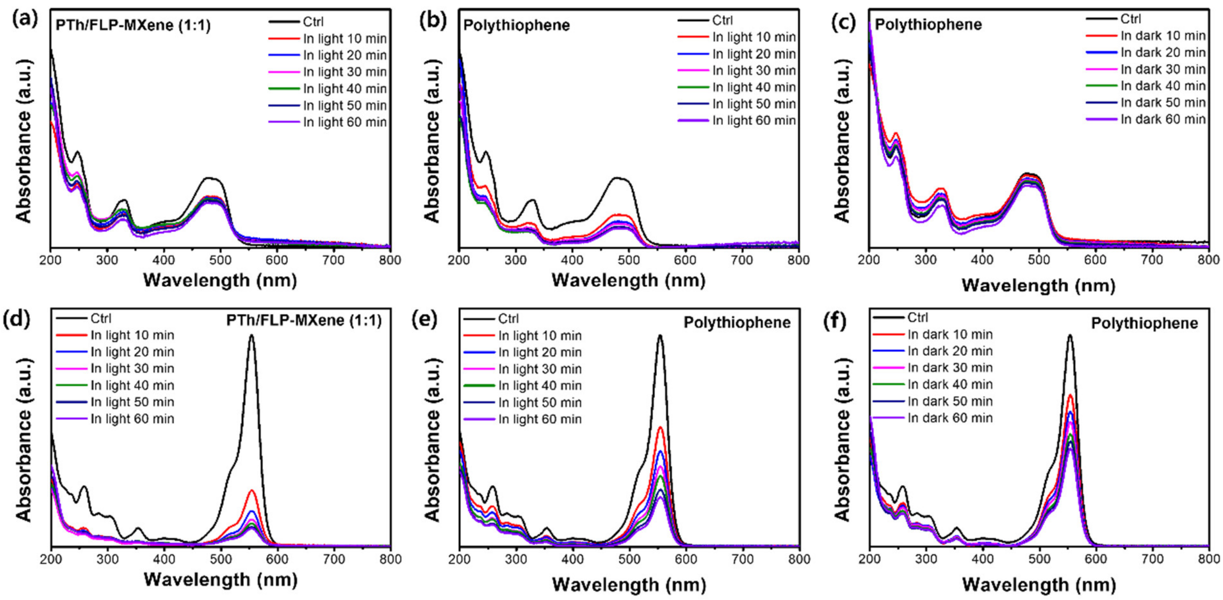

Figure S9. (a-c) Time-dependent UV-Vis absorption spectra of OG solutions containing PTh/FLP MXene composite (1:1) and PTh nanospheres in light or dark conditions. (d-f) Time-dependent UV-Vis absorption spectra of RhB solutions containing PTh/FLP MXene composite (1:1) and PTh nanospheres in light or dark conditions. For (a) and (d), PTh/FLP MXene composite (1:1) was used while PTh nanospheres were used for the others.

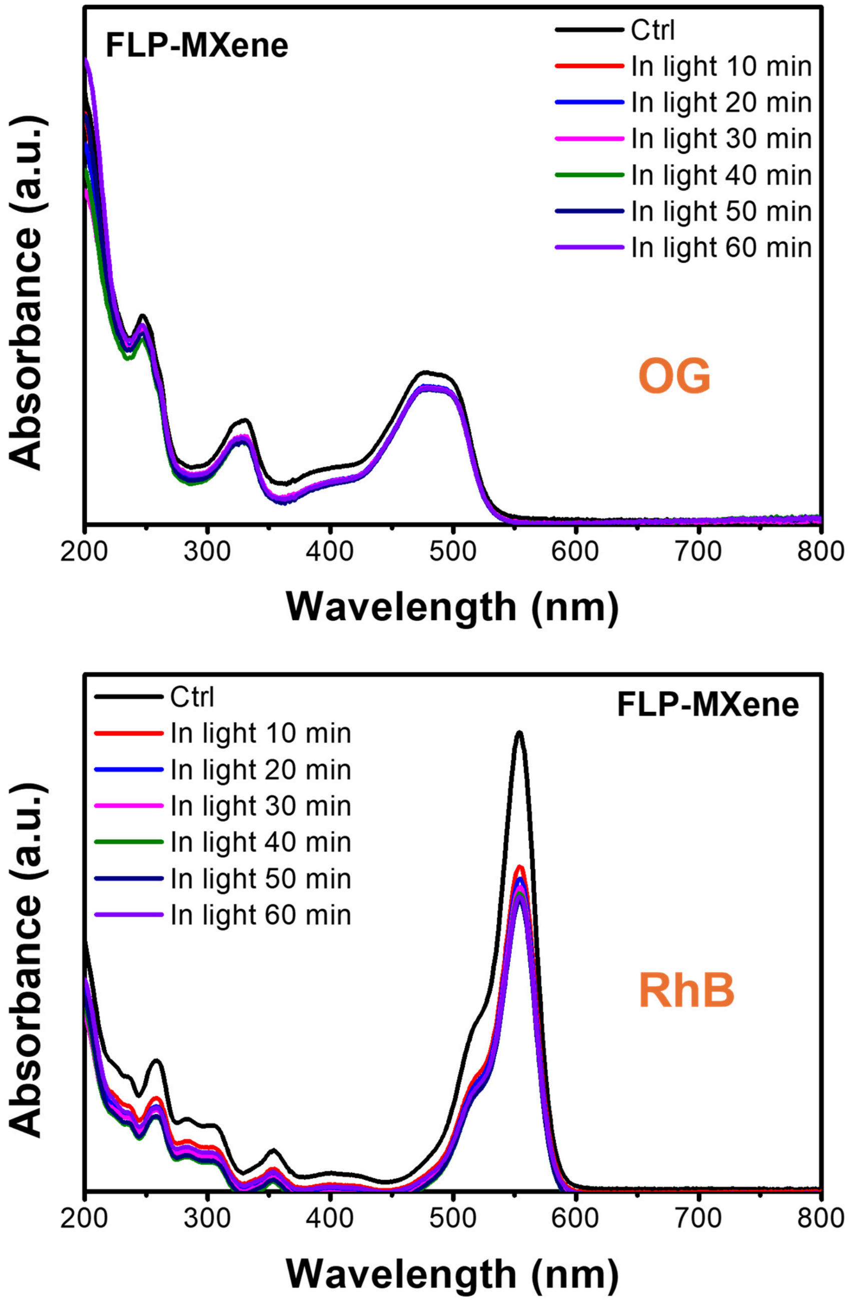

Figure S10. Time-dependent UV-Vis absorption spectra of (a) OG and (b) RhB solutions. Here, FLP MXene was used as a photocatalyst.

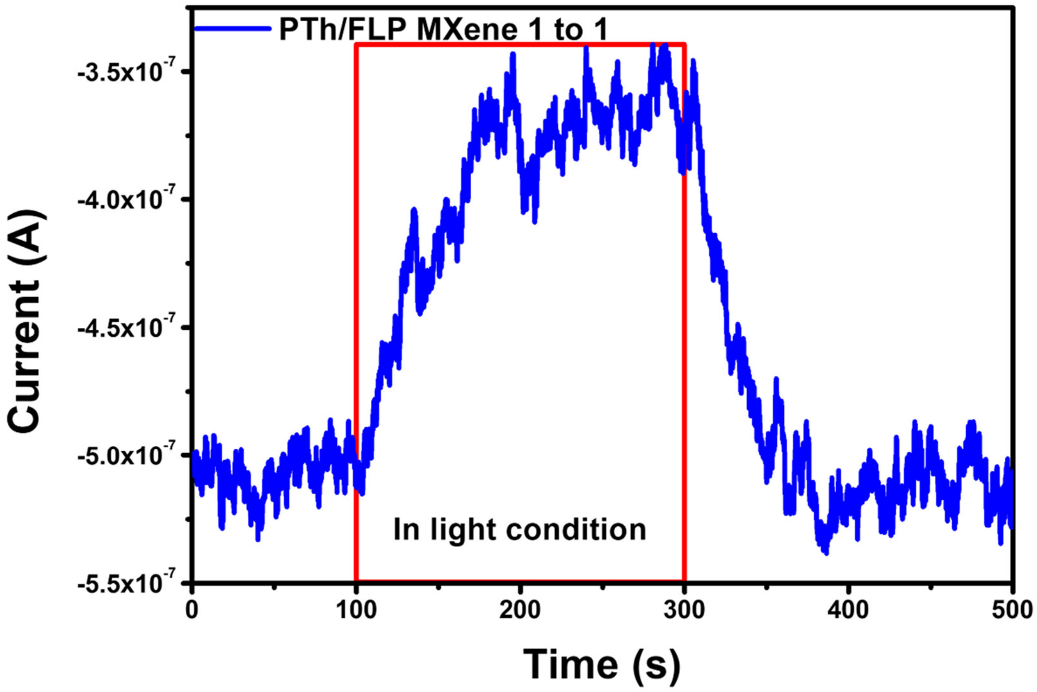

Figure S11. Photocurrent response of PTh/FLP MXene composite (1:1).
